# Prenatal phenotypes and pregnancy outcomes of fetuses with recurrent 1q21.1 microdeletions and microduplications

**DOI:** 10.3389/fmed.2023.1207891

**Published:** 2023-08-24

**Authors:** Fagui Yue, Xiao Yang, Yuting Jiang, Shibo Li, Ruizhi Liu, Hongguo Zhang

**Affiliations:** ^1^Center for Reproductive Medicine and Center for Prenatal Diagnosis, First Hospital, Jilin University, Changchun, China; ^2^Jilin Engineering Research Center for Reproductive Medicine and Genetics, Jilin University, Changchun, China; ^3^Department of Pediatrics, University of Oklahoma Health Sciences Center, Oklahoma City, OK, United States

**Keywords:** chromosomal 1q21.1 microdeletions and microduplications, chromosomal microarray analysis, prenatal phenotypes, pregnancy outcomes, cerebral ventriculomegaly

## Abstract

**Objective:**

Chromosomal 1q21.1 deletions and duplications are genomic disorders that are usually diagnosed postnatally. However, the genotype–phenotype correlations of 1q21.1 copy number variants (CNVs) during the prenatal period are still not clear. This study aimed to provide a systematic summary of prenatal phenotypes for such genomic disorders.

**Methods:**

In total, 26 prenatal amniotic fluid samples diagnosed with 1q21.1 microdeletions/microduplications were obtained from pregnant women who opted for invasive prenatal testing. Karyotypic analysis and chromosomal microarray analysis (CMA) were performed for all cases simultaneously. The pregnancy outcomes and health conditions after birth in all cases were followed up. Meanwhile, prenatal cases with 1q21.1 microdeletions or microduplications in the literature were retrospectively collected.

**Results:**

In total, 11 pregnancies (11/8,252, 0.13%) with 1q21.1 microdeletions and 15 (15/8,252, 0.18%) with 1q21.1 microduplications were identified. Among these 1q21.1 CNVs, 4 cases covered the thrombocytopenia-absent radius (TAR) region, 16 cases covered the 1q21.1 recurrent microdeletion/microduplication region, and 6 cases covered all regions mentioned above. The prenatal abnormal ultrasound findings were recorded in four participants with 1q21.1 deletions and seven participants with 1q21.1 duplications. Finally, three cases with 1q21.1 deletions and five with 1q21.1 duplications terminated their pregnancies.

**Conclusion:**

In the prenatal setting, 1q21.1 microdeletions were associated with increased nuchal translucency (NT), anomalies of the urinary system, and cardiovascular abnormalities, while 1q21.1 microduplications were correlated with cardiovascular malformations, nasal bone dysplasia, and increased NT. In addition, cerebral ventriculomegaly might be correlated with 1q21.1 microduplications. Considering the variable expressivity and incomplete penetrance of 1q21.1 CNVs, long-term follow-up after birth should be carried out in these cases.

## 1. Introduction

Chromosomal rearrangements involving the 1q21.1 region are hotspot loci that are frequently discovered in patients with different clinical manifestations. The multiple low-copy repeats located in chromosome 1q21.1 could make this region susceptible to non-allelic homologous recombination (NAHR), which would cause recurrent deletions and duplications (1–3). Four segmental duplication blocks, referred to as breakpoints (BPs) BP1–BP4, were specified within the 1q21.1 region from centromere to telomere (4). The chromosomal 1q21.1 region is usually subdivided into two distinctive regions: the proximal region extends from BP2 to BP3, spanning ~0.2 Mb (chr1: 145.4–145.6 Mb, GRCh37/hg19), and the distal region extends from BP3 to BP4, spanning ~1.35 Mb (chr1: 146.5–147.9 Mb, GRCh37/h19) (1, 2). In addition, two classes, 1q21.1 deletions and duplications, were defined at the molecular level: class I located between BP3 and BP4 (~1.8 Mb) and class II located between BP1/BP2 and BP4 (~2.7 Mb) (3, 4).

**Figure 1:**
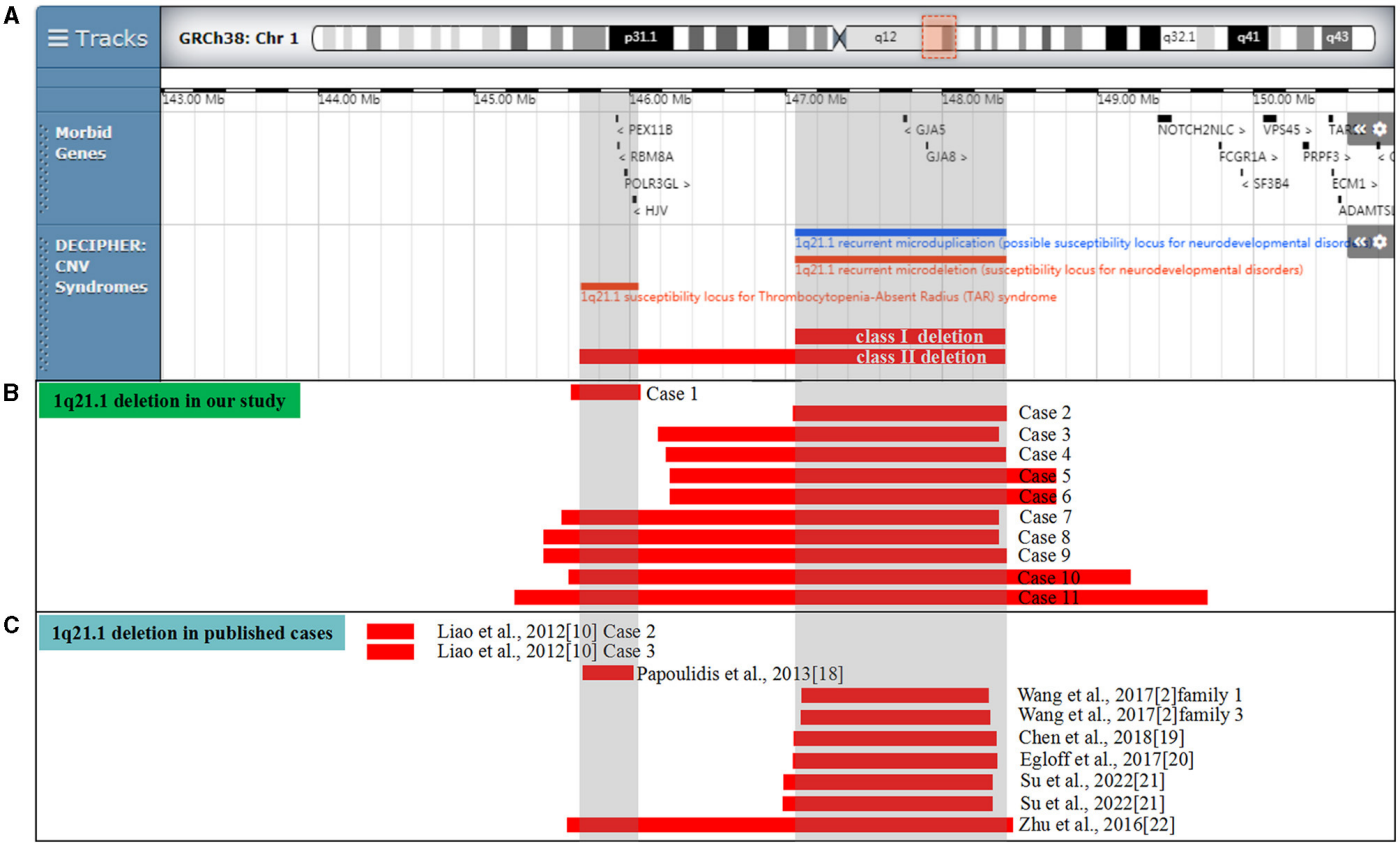
Scale representation of the deleted region in the 1q21.1 region (https://decipher.sanger.ac.uk/): **(A)** location of genes and genomic syndromes in the region; **(B)** deleted fragments in the present cases; and **(C)** previously described 1q21.1 deletions in the prenatal period. Genomic parameters are from GRCh38/hg38.

**Figure 2:**
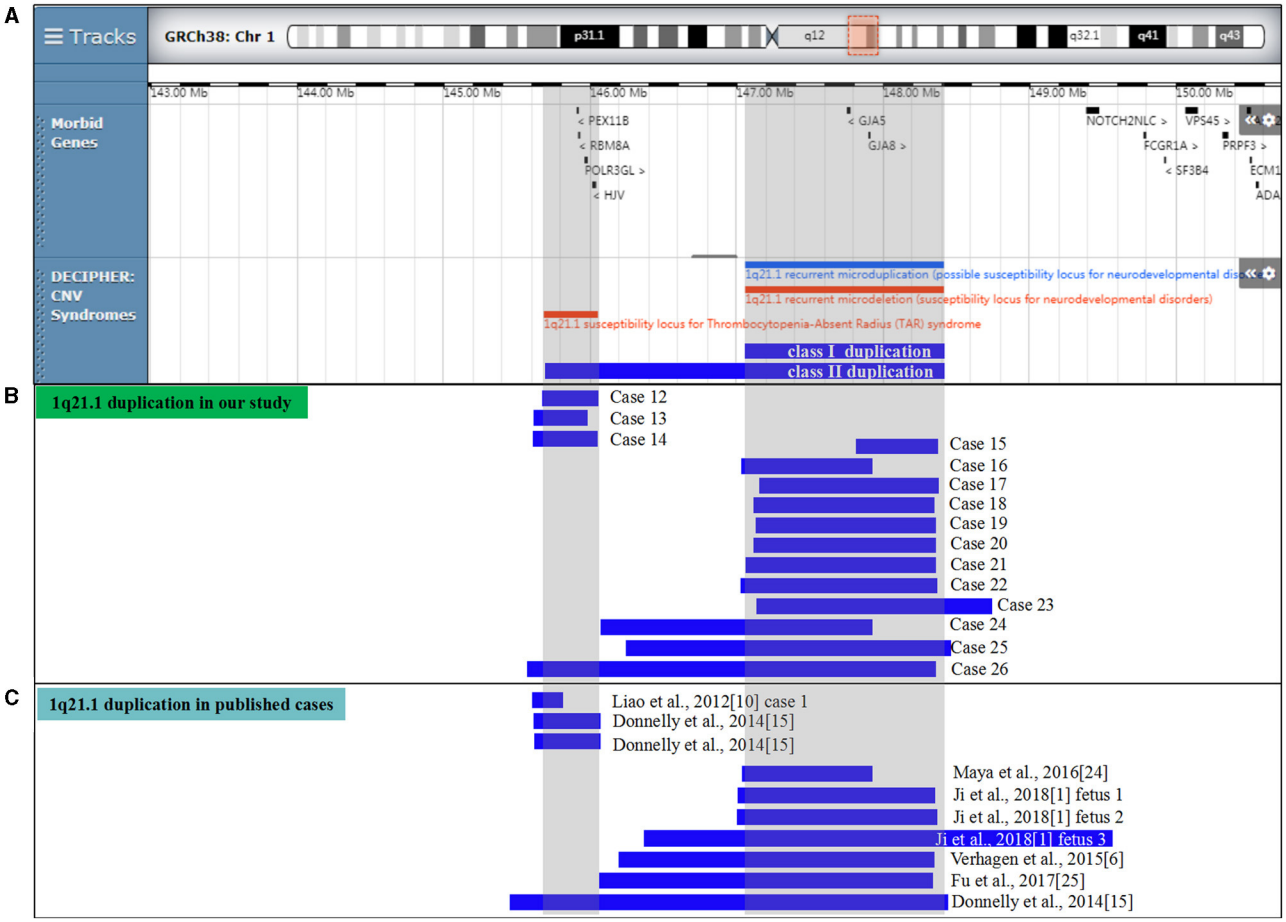
Scale representation of the duplicated region in the 1q21.1 region (https://decipher.sanger.ac.uk/): **(A)** location of genes and genomic syndromes in the region; **(B)** duplicated fragments in the present cases; and **(C)** previously described 1q21.1 duplications in the prenatal period. Genomic parameters are from GRCh38/hg38.

Three clinic disorders with diverse copy number variants (CNVs) within the 1q21.1 region were described: chromosome 1q21.1 deletion syndrome (OMIM 612474), chromosome 1q21.1 duplication syndrome (OMIM 612475), and thrombocytopenia-absent radius (TAR) syndrome (OMIM 274000) (Figures 1A, 2A). Chromosome 1q21.1 deletions have been associated with developmental delay, intellectual disability, autism spectrum disorders, attention deficit hyperactivity disorder (ADHD), schizophrenia, cataracts, dysmorphic features (microcephaly, frontal bossing, deep-set eyes, epicanthic folds, large nasal bridge, long philtrum, highly arched palate, and trigonocephaly), and congenital anomalies (congenital heart disease, eye abnormalities, skeletal, and genitourinary malformations). Chromosomal 1q21.1 duplications exhibit a wide spectrum of anomalies, including developmental delay, intellectual disability, autism spectrum disorder, macrocephaly, congenital heart anomalies, and dysmorphic features (e.g., frontal bossing and hypertelorism) (5–7). The TAR syndrome is recognized as a congenital malformation syndrome characterized by bilateral absence of the radii and thrombocytopenia, musculoskeletal and gastrointestinal abnormalities, renal and cardiac anomalies, and intolerance to cow's milk (8, 9). It is evident that the clinical manifestations of CNVs at the 1q21.1 region are diverse and complicated, some of which could not be identified even by advanced machines and experienced clinicians in ultrasound.

Till now, most studies on the CNV spectrum in the 1q21.1 region were diagnosed postnatally. Prenatal reports involving 1q21.1 duplications and deletions were limited (6, 10). To better our understanding of these prenatally detected chromosomal microscopic imbalances, we present the clinical and molecular findings of 26 cases with 1q21.1 microdeletions and microduplications in pregnant women undergoing prenatal invasive testing and provide a systematic summary of prenatal phenotypes for such genomic disorders.

## 2. Materials and methods

### 2.1. Clinical data

This retrospective study was performed from October 2018 to November 2022 and enrolled 26 cases carrying 1q21.1 microdeletions and microduplications, which were selected from 8,252 pregnant women. These women were referred to the First Hospital of Jilin University and underwent invasive diagnostic testing via amniocentesis. The main indications for prenatal diagnosis included non-invasive prenatal testing (NIPT) for aneuploidy, serum screening results for aneuploidy, ultrasound anomalies, and advanced maternal age. All pregnant women accepted routine prenatal ultrasound examinations during the gestation period, and abnormal ultrasound findings were included in the indications for prenatal diagnosis. All couples denied consanguineous marriage, and the pregnant women denied any exposure to teratogenic agents, irradiation, or infectious diseases during the pregnancy in question. All the prospective parents received detailed genetic counseling, and blood samples were collected after obtaining informed consent. The study protocol was approved by the Ethics Committee of the First Hospital of Jilin University (No. 2021-706), and written informed consent was obtained from all the couples.

### 2.2. Cytogenetic analysis

Pregnant women accepted amniocentesis for karyotyping analysis with written informed consent. A total of 30 ml of amniotic fluid cells were collected. Routine cytogenetic analysis was performed on G-band metaphases at 400–500 banding resolution, which was prepared from 20 ml of cultured amniotic fluid cells in accordance with standard protocols in our lab. In total, 20 metaphases were analyzed for all samples.

### 2.3. Chromosomal microarray analysis

The genomic DNA was extracted from the amniotic fluid cells and parental peripheral blood with the QIAamp^®^ DNA Blood Mini Kit (Qiagen, Inc., Hilden, Germany) according to the manufacturer's protocol. Following written consent from all pregnant women, 10 ml of uncultured amniotic fluid cells were collected through amniocentesis. Then, the procedures were conducted through the CytoScan 750K array (Affymetrix, Santa Clara, CA, USA), in accordance with the manufacturer's protocol and our previous study (11). The procedure included genomic DNA extraction, digestion and ligation, PCR amplification, PCR product purification, quantification and fragmentation, labeling, array hybridization, washing, and scanning. Thresholds for genome-wide screening were set at ≥100 kb for gains and losses. The detected CNVs were comprehensively estimated by comparing them with the published literature and the public databases: (1) Database of Genomic Variants (DGVs; http://dgv.tcag.ca/dgv/app/home), (2) Database of Chromosomal Imbalance and Phenotype in Humans using Ensemble Resources (DECIPHERs, http://decipher.sanger.ac.uk/), (3) International Standards for Cytogenomic Arrays (ISCAs; https://www.iscaconsortium.org/), (4) Online Mendelian Inheritance in Man (OMIM, http://www.ncbi.nlm.nih.gov/omim), and (5) UCSC (http://genome.ucsc.edu/). All CNVs were classified as pathogenic (P), likely pathogenic (LP), variants of unknown significance (VOUS), likely benign (LB), and benign (B). Genomic positions refer to the Human Genome Assembly Dec. 2013 (GRCh38/hg38).

### 2.4. Selection of prenatally detected 1q21.1 microdeletion and microduplication

Given the lack of prenatal phenotypes of 1q21.1 deletions and duplications reported in the literature, we launched a search on PubMed (https://www.ncbi.nlm.nih.gov/pubmed/) for identifying relevant articles from inception to 2022. Criteria for case selection were defined as being in the English language, 1q21.1 deletions and duplications, and ultrasound phenotypes. Meanwhile, in order to investigate the candidate genes related to abnormal phenotypes, chromosomal microarray results for all reviewed cases should be guaranteed. A string of the following terms and their synonyms were used: 1q21.1 deletion/loss, 1q21.1 duplication/gain, prenatal diagnosis, chromosomal microarray analysis, and ultrasound findings. The combination of subject words and free words was also used for the search. The information collected included the general condition of the subjects (age, gravida and para, gestational age, and parental phenotypes), karyotype, inheritance, microarray results, indications for prenatal diagnosis (including ultrasound findings), and pregnancy outcomes.

### 2.5. Follow-up outcomes

The follow-up was mainly carried out through telephone interviews using a customized questionnaire by our center's follow-up staff after all the women had given birth. The specific follow-up contents contained pregnancy outcomes (miscarriages or birth), gestational ages of delivery, sex and birth weight/length of the neonate, ultrasound findings during the pregnancy period (nervous system, cardiovascular system, craniofacial growth, respiratory system, abdominal abnormalities, urinary system, alimentary system, musculoskeletal system, and others), and postnatal health conditions (congenital defects, craniofacial dysmorphisms, skeletal anomalies, and developmental details).

## 3. Results

### 3.1. Study population

Of 8,252 pregnant women opting for prenatal invasive testing in our center, 11 fetuses were identified with 1q21.1 microdeletions and 15 were diagnosed with 1q21.1 microduplications. The detection rate of CNVs involving the chromosomal 1q21.1 region was 0.31% (26/8,252). Tables 1, 2 summarize the clinical information, including gestational week at detection, indications for prenatal diagnosis, CMA results, maternal inheritance, and pregnancy outcomes for all the cases.

**Table 1:** Summary of clinical and molecular findings of fetuses presenting 1q21.1 microdeletion detected by CMA.

**Our case No**.	**Age**	**Gravida and para**	**Gestational age (weeks)**	**Indications for prenatal diagnosis**	**Parental phenotypes**	**Region**	**Karyotype**	**CMA results (GRCh38)**	**Size (Mb)**	**Inheritance**	**Morbid genes**	**Pathogenicity**	**Pregnancy outcome**		
													**Gestational age**	**Length (cm)**	**Birth weight (kg)**
1	27	G1P0	19+	Increased NT (4.5 mm)	Normal	1q21.1	46,XN	1q21.1 (145605589-146061433) × 1	0.45	*de novo*	PEX11B, RBM8A, POLR3GL, HJV	LP	TOP at 25 w		
2	20	G1P0	19+	Mother:1q21.1 deletion carrier	Mother: optic atrophy and retinal detachment Father: difficulty in moving, normal intelligence	1q21.1q21.2	46,XN	1q21.1q21.2 (147032846-148413447) × 1	1.38	Mat	GJA5, GJA8	P	TOP at 23 w 1d		
3	31	G1P0	16+	NIPT infers a high risk of chromosome 16; fetal growth restriction and microcephaly (35 w 6 d)	Normal	1q21.1q21.2	46,XN	1q21.1q21.2 (146174424-148358701) × 1	2.63	Mat	GJA5, GJA8	P	38 w	49	2.8
4	31	G3P2	18+	Increased NT (3.1 mm), abnormal childbearing history (boy: developmentally delay and intellectual disability presenting 46,XY, t(1;6) (p22;q21) and 1q21.1 microdeletion	Normal (mother: 46,XX father: 46,XY)	1q21.1q21.2	46,XN	1q21.1q21.2 (146209793-148413447) × 1	2.2	*de novo*	GJA5, GJA8	P	TOP at 23 w		
5	26	G1P0	17+	NIPT infers a high risk of chromosome 9	Normal	1q21.1q21.2	46,XN	1q21.1q21.2 (146242158-148731429) × 1	2.48	n.a.	GJA5, GJA8	P	39 w 6 d	51	3.7
6	28	G1P0	29+	Aberrant right subclavian artery; ventricular apical thin point	Normal	1q21.1q21.2	46,XN	1q21.1q21.2 (146256254-148731429) × 1	2.47	n.a.	GJA5, GJA8	P	40 w 5 d	50	3.6
7	24	G1P0	18+	Risk of fetal trisomy 21: 1/53	Normal	1q21.1q21.2 16q23.1	46,XN	1q21.1q21.2 (145568752-148358701) × 1 16q23.1 (75491006-75655382) × 3	1.80 0.16	n.a.	PEX11B, RBM8A, POLR3GL, HJV, GJA5 GJA8	P VOUS	TOP		
8	41	G3P0A1	18+	AMA	Normal	1q21.1q21.2 16p11.2	46,XN	1q21.1q21.2 (145430996-148358701) × 1 16p11.2 (32513443-34061205) × 3	1.93 1.33	*de novo de novo*	PEX11B, RBM8A, POLR3GL, HJV, GJA5, GJA8	LP LB	35 w	48	2.5
9	30	G2P0	18+	Abnormal childbearing history (a fetus presenting VSD with maternally inherited 1q21.1 deletion (TOP at 37 w)	Normal	1q21.1q21.2	46,XN	1q21.1q21.2 (145430996-148413447) × 1	1.98	Mat	PEX11B, RBM8A, POLR3GL, HJV, GJA5, GJA8	P	37 w	48	2.8
10	42	G1P0	22+	AMA, increased NT	Normal	1q21.1	46,XN	1q21.1 (145601946-149194711) × 1	1.39	Mat	PEX11B, RBM8A, POLR3GL,HJV, GJA5, GJA8	LP	39 w 2 d	50	2.75
11	18	G1P0	28+	NIPT infers a high risk of chromosome 1	Normal	1q21.1q21.2	46,XN	1q21.1q21.2 (145264933-149704737) × 1	4.43	Mat	PEX11B, RBM8A, POLR3GL, HJV, GJA5 GJA8, NOTCH2NLC	P	TOP at 30 w		

AMA, advanced maternal age; CMA, chromosomal microarray analysis; d, days; w, weeks; LB, likely benign; LP, likely pathogenic; mat, maternal; n.a., not available; NIPT, non-invasive prenatal testing; NT, nuchal translucency; pat, paternal; P, pathogenic; TOP, termination of pregnancy; VOUS, variants of unknown significance; VSD, ventricular septal defect.

**Table 2:** Summary of clinical and molecular findings of fetuses presenting 1q21.1 microduplication detected by CMA.

**Our case No**.	**Age**	**Gravida and para**	**Gestational age (weeks)**	**Indications for prenatal diagnosis**	**Parental phenotypes**	**Region**	**Karyotype**	**CMA results (GRCh38)**	**Size (Mb)**	**Inheritance**	**Morbid genes**	**Pathogenicity**	**Pregnancy outcome**		
													**Gestational age**	**Length (cm)**	**Birth weight (g)**
12	37	G3P1	19+	AMA	Normal	1q21.1	46,XN	1q21.1 (145670380-146044897) × 3	0.37	Mat	PEX11B, RBM8A, POLR3GL, HJV,	VOUS	39 w 4 d	50	3.4
13	32	G2P0	25+	Abnormal childbearing history (child with cardiac malformation)	Mother: Intellectual disability	1q21.1	46,XN	1q21.1 (145605588-145966247) × 3	0.36	*de novo*	PEX11B, RBM8A, POLR3GL	VOUS	38 w 5 d	50	3.05
14	38	G2P1	18+	AMA, prenatal ultrasound findings infer cerebral ventriculomegaly	Normal	1q21.1	46,XN	1q21.1 (145605589-146044871) × 3	0.43	n.a.	PEX11B, RBM8A, POLR3GL, HJV	VOUS	39 w	50	3.6
15	31	G3P1	24+	Abnormal childbearing history (child presenting language retardation)	Normal	1q21.2 3p12.3	46,XN	1q21.2 (1:147800251-148372635) × 3 3p12.3 (74691013-75715264) × 3	0.57 1.02	mat *de novo*	GJA8	LP LB	39 w 1 d	50	3.25
16	28	G1P0	18+	Increased NT, absence of nasal bone	Normal	1q21.1q21.2 7q36.1	46,XN	1q21.1q21.2 (147024824-147921222) × 3 7q36.1 (152011707-152398273) × 3	1.36 0.38	pat *de novo*	GJA5,GJA8	P VOUS	TOP at 25 w		
17	29	G2P0	19+	Prenatal ultrasound findings infer VSD and the absence of nasal bone	Normal	1q21.1q21.2	46,XN	1q21.1q21.2 (147131352-148372635) × 3	1.24	Pat	GJA5,GJA8	P	41 w	51	3.7
18	35	G2P1	23+	AMA, abnormal childbearing history (trisomy 21)	Normal	1q21.1q21.2	46,XN	1q21.1q21.2 (147114667-148342369) × 3	1.22	Mat	GJA5,GJA8	P	38 w 6 d	50	3.5
19	26	G1P0	17+	Voluntary request, no abnormal ultrasound findings observed	Normal	1q21.1q21.2	46,XN	1q21.1q21.2 (147132973-148358701) × 3	1.22	*de novo*	GJA5, GJA8	P	TOP at 27 w		
20	34	G2P1	28+	Prenatal ultrasound findings infer cerebral ventriculomegaly	Normal	1q21.1q21.2	46,XN	1q21.1q21.2 (147111142-148358701) × 3	1.24	n.a.	GJA5, GJA8	P	TOP		
21	28	G1P0	18+	Father: 46,XY, inv(6) (p21.1q25)	Father: teratospermia	1q21.1q21.2	46,XN, inv(6) (p21.1q25)	1q21.1q21.2 (147056729-148358701) × 3	1.30	*de novo*	GJA5, GJA8	P	TOP at 23 w 2 d		
22	37	G1P0	19+	AMA	Normal	1q21.1q21.2	46,XN	1q21.1q21.2 (147016573-148358701) × 3	1.34	Pat	GJA5, GJA8	P	39 w	50	3.5
23	33	G2P0	20+	Increased NT	Normal	1q21.1q21.2	46,XN	1q21.1q21.2 (147115536-148742984) × 3	1.62	Pat	GJA5, GJA8	P	39 w 2 d	55	4.75
24	35	G3P1	19+	AMA, abnormal childbearing history (child presenting cerebral palsy, developmental delay, and scoliosis)	Normal	1q21.1q21.2	46,XN	1q21.1q21.2 (146066001-147919795) × 3	1.28	n.a.	GJA5, GJA8	P	TOP at 22 w		
25	45	G3P1	24+	AMA, tetralogy of fallot	Normal	1q21.1q21.2	46,XN	1q21.1q21.2 (146234373-148456994) × 4	2.22	n.a.	GJA5,GJA8	P	39 w	50	3.6
26	23	G2P1	17+	Prenatal ultrasound findings infer short nasal bone	Normal	1q21.1q21.2 8p23.3	46,XN	1q21.1q21.2 (145568752-148348214) × 3 8p23.3 (208048-1410532) × 1	1.79 1.2	n.a.	PEX11B, RBM8A, POLR3GL, HJV GJA5, GJA8	P VOUS	TOP at 32 w due to brain anomalies		

AMA, advanced maternal age; CMA, chromosomal microarray analysis; d, days; w, weeks; LB, likely benign; LP, likely pathogenic; mat, maternal; n.a., not available; NIPT, non-invasive prenatal testing; NT, nuchal translucency; pat, paternal; P, pathogenic; TOP, termination of pregnancy; VOUS, variants of unknown significance; VSD, ventricular septal defect.

### 3.2. Chromosomal anomalies detected by karyotyping

Amniotic fluid samples from all subjects were subjected to conventional karyotyping to identify balanced chromosomal rearrangements that could not be detected through CMA. Of the 11 1q21.1 microdeletions, no karyotypic anomalies were observed. Among the 15 1q21.1 microduplications, the karyotype of case 21 was 46,XN,inv(6) (p21.1q25).

### 3.3. Chromosome 1q21.1 microdeletions in affected fetuses

In our report, 11 cases (0.13%, 11/8,252) with 1q21.1 microdeletions were identified by CMA, ranging from 0.45 Mb to 4.43 Mb (Table 1). In addition, CMA detected a 0.16 Mb duplication of 16q23.1 in case 6 and a 1.33 Mb duplication of 16p11.2 in case 8, the clinic pathogenicity of which was VOUS and LB, respectively. The distribution of indications for prenatal diagnosis was as follows: non-structural anomalies (5/11), NIPT inferring aneuploidy (3/11), abnormal childbearing history (2/11), advanced maternal age (2/11), risk of Down syndrome (1/11), and maternal abnormal karyotype (1/11). The abnormal ultrasound findings were recorded in five participants with 1q21.1 deletions, three of whom presented increased nuchal translucency (NT). Among the 5 of 11 cases with maternal inheritance, only the mother of case 2 presented optic atrophy and retinal detachment. Three of the 11 cases were *de novo*, and 3 of the 11 cases were not available. One case (case 1) encompassed the TAR region, five cases (cases 2–6) encompassed 1q21.1 recurrent microdeletion, and five cases (cases 7–11) covered both the TAR region and 1q21.1 recurrent microdeletion in common (Figure 1B).

### 3.4. Chromosome 1q21.1 microduplications in affected fetuses

CMA successfully identified 15 fetuses (0.18%, 15/8,252) with 1q21.1 microduplications ranging from 0.36 Mb to 1.79 Mb (Table 2). In addition, CMA detected a 0.57 Mb duplication of 3p12.3 in case 15, a 0.38 Mb duplication of 7q36.1 in case 16, and a 1.2 Mb deletion of 8p23.2 in case 26, with LB, VOUS, and VOUS clinic pathogenicity, respectively. The distribution of indications for prenatal diagnosis was as follows: advanced maternal age (6/15), nasal bone dysplasia (3/15), cerebral ventriculomegaly (2/15), cardiac malformation (2/15), increased NT (2/15), abnormal childbearing history (4/15), voluntary request (1/15), and parental chromosome anomaly (1/15). Prenatal abnormal ultrasound findings were recorded in seven cases, in which cerebral ventriculomegaly, nasal bone dysplasia, and heart malformations were observed. Among them, 7 of 15 cases were parentally inherited, 3 of 15 cases were *de novo*, and 5 of 15 cases were unavailable. Three cases (cases 12–14) encompassed the TAR locus, 11 cases (cases 15–25) shared 1q21.1 recurrent microduplication, and 1 case (case 26) covered the TAR region and 1q21.1 recurrent microduplication (Figure 2B).

### 3.5. Prenatal and postnatal follow-up assessment

Of the 11 1q21.1 deletion cases, five eventually terminated their pregnancies: two (cases 1 and 4) were *de novo*, two (cases 2 and 11) were maternally inherited, and one (case 7) was unavailable. Among the six cases that continued the pregnancies, three were maternal inheritance, one was *de novo*, and two were unavailable. It was noteworthy that the two pregnancies of case 9 carried the maternally inherited 1q21.1 deletion. However, the first pregnancy presented a ventricular septal defect (VSD), while no ultrasound findings were discovered for her second pregnancy, so the woman continued the pregnancy and delivered a healthy child at term. In the 15 1q21.1 duplication cases, 6 opted for the termination of pregnancy (TOP): 2 cases (cases 19 and 21) were *de novo*, 1 (case 16) was paternally inherited, and 3 (cases 20, 24, and 26) were unavailable. Among the nine cases opting for ongoing pregnancies, six were parentally inherited, one was *de novo*, and two were unavailable. The clinic pathogenicity of the duplicated TAR region was VOUS, which was probably the reason for ongoing pregnancies in cases 10–12.

We followed up on all neonates with 1q21.1 microdeletions and microduplications after birth, including congenital defects, craniofacial dysmorphisms, and skeletal anomalies developmental details. Overall, they were in healthy states, with no evident anomalies observed up until the writing of this article. However, since all subjects were of a young age, long-term follow-up should be guaranteed for them.

## 4. Discussion

In our study, we systematically described 26 prenatal cases referred to our center for prenatal invasive testing and found recurrent chromosomal 1q21.1 rearrangements. Among them, chromosomal 1q21.1 microdeletions were detected in 11 cases, five of which eventually chose TOP. Chromosomal 1q21.1 microduplications were identified in 15 cases, and six opted for TOP. Compared with postnatal phenotypes, prenatal phenotypes involving 1q21.1 deletions/duplications were limited in the clinic. To the best of our knowledge, this is the largest cohort study with a detailed follow-up for prenatally diagnosed CNVs at the 1q21.1 locus in China.

CMA has been adopted as an effective diagnostic tool in identifying new microdeletion and microduplication syndromes, such as Williams–Beuren syndrome, 17q21.31 microdeletion syndrome, Prader–Willi syndrome, and Angelman syndrome. As a hot spot region, CNVs at the 1q21 locus were frequently reported in postnatal settings and in populations with intellectual disabilities, developmental delays, schizophrenia, and autism (12). In a study involving 5,218 persons with idiopathic intellectual disabilities, autism, or congenital anomalies, Mefford et al. identified 25 unrelated probands with 1q21.1 deletions (0.5%) and nine persons with 1q21.1 duplications (0.2%), with no 1q21.1 microdeletions and only one microduplication found in 4,737 controls (13). Brunetti-Pierri et al. described 21 probands with 1q21.1 microdeletions and 15 probands with 1q21.1 microduplications in 16,557 affected individuals presenting intellectual disabilities, autism, and/or congenital anomalies, with detection frequencies of 0.13 and 0.09%, respectively (14). However, the detection rates of CNVs at the 1q21.1 locus in a prenatal setting were rarely described. It was reported that the frequencies of chromosomal 1q21.1 deletions/duplications with and without fetal anomalies were 4.9 and 9.6%, respectively (15, 16). In our 8,252 prenatal cases referred for genetic microarray testing, the detection rates of 1q21.1 microdeletions and microduplications were 0.13 and 0.18%, respectively.

As one of the most commonly detected structural aberrations, individuals carrying chromosomal 1q21.1 CNVs could exhibit diverse phenotypes, including intellectual disabilities, autism, schizophrenia, congenital anomalies, dysmorphic features, or normal phenotypes (17). Till now, most research involving 1q21.1 CNVs focused on postnatal cases, and the prenatal genotype–phenotype correlation was still unclear due to inadequate reports in the clinic. Considering the phenotypic diversity, the incomplete penetrance, and the lack of distinct prenatal features for 1q21.1 CNVs, it is challenging to offer genetic counseling for such prenatal cases. Hence, to provide a better understanding of 1q21.1 CNVs in the prenatal setting, we summarized the clinical data and molecular findings of prenatally detected 1q21.1 microdeletions and microduplications in the published literature (Tables 3, 4, Figures 1C, 2C).

**Table 3:** Clinical data of fetuses presenting 1q21.1 microdeletion detected by CMA in the published literature.

**No**.	**Age**	**Gravida and para**	**Gestational age (weeks)**	**Karyotype**	**Deleted region**	**Deleted Size**	**Inheritance**	**CMA results (GRCh38)**	**Referred critical gene**	**Prenatal ultrasound findings**	**Pregnancy outcome**	**References**
1	27	G1P0	20+	46,XX	1q21.1	317 kb	*de novo*	1q21.1 (145601946-145853772) × 1	PDZK1, GPR89A, CD160, RNF115, GPR89C	Right kidney absent, megalo-ureter; oligohydramnios; single umbilical artery	Not referred	(10) case 2
2	28	G2P0	16+	46,XX	1q21.1	317 kb	Pat	1q21.1 (145601946-145853772) × 1	PDZK1, GPR89A, CD160, RNF115, GPR89C	Bilateral renal dysplasia, almost no amniotic fluid; bladder not visible (the father presenting polycystic right kidney)	Ongoing pregnancy	(10) case 3
3	30	G2P1	22+	46,XX	1q21.1q21.2	1.317Mb	Pat	1q21.1q21.2 (147035964-148352079) × 1	RKAB2, FMO5, CHD1L, BCL9, ACP6, GJA5, GJA8, GPR89B	Fetal polydactyly of the left foot and echogenic heart foci	3,416 g female baby with postaxial polydactyly of the left foot.	(19)
4	n.a.	n.a.	n.a.	n.a.	1q21.1q21.2	1.89 Mb	n.a.	1q21.1q21.2 (145583523-148457202) × 1	PEX11B, RBM8A, POLR3GL, HJV, GJA5, GJA8	VSD	Not referred	(22)
5	33	n.a.	13+	n.a.	1q21.1	1.3 Mb	*de novo*	1q21.1 (147034756-148352079) × 1	GJA5, GJA8	NT = 4.9 mm	Live birth	(20)
6	29	G3P1	13+	46,XY	1q21.1	334 kb	Pat	1q21.1 (145,413,388-145,747,269) × 1	RBM8A	NT = 8.1 mm	TOP at 14 w	(18)
7	26	G2P1	n.a.	46,XN	1q21.1	1.22 Mb	Mat	1q21.1 (147093177-148314590) × 1	GJA5, GJA8	Encephalomeningocele	TOP	(2) family 1
8	28	G1P0	n.a.	46,XN	1q21.1	1.22 Mb	*de novo*	1q21.1 (147093177-148314590) × 1	GJA5, GJA8	Complete atrioventricular septal defect	TOP	(2) family 3
9–10	27–34	n.a.	23–25	n.a.	1q21.1	1.3 Mb	Inherited/ unknown (1/1)	1q21.1 (146964802-148327911) × 1	GJA5	Multicystic dysplastic kidney (1), ectopic kidney (1)	Live birth (2)	(21)

CMA, chromosomal microarray analysis; mat, maternal; n.a., not available; NT, nuchal translucency; pat, paternal; w, weeks; TOP, termination of pregnancy; VSD, ventricular septal defect.

**Table 4:** Pooled data from all fetuses presenting 1q21.1 microdeletion and microduplication.

**CNVs**	**1q21.1 microdeletion**		**1q21.1 microduplication**	
	**Previous reports**	**Our study**	**Previous reports**	**Our study**
Age (y)	29 (26–34)	29 (18–42)	28 (23–32)	33 (23–45)
Gestational age (w)	19 (13–25)	20 (16–29)	21 (11–27)	21 (17–28)
Ultrasound findings				
Cardiovascular abnormalities	2/10	1/11	5/10	2/15
Increased NT	2/10	3/11	2/10	2/15
Anomalies of the urinary system	4/10		2/10	
Nasal bone dysplasia			2/10	3/15
Skeletal dysplasia	1/10		1/10	
Nervous system abnormalities	1/10	1/11		
Oligohydramnios	2/10		2/10	
Cerebral ventriculomegaly				2/15
Fetal growth restriction		1/11		
Aberrant right subclavian artery		1/11		
Ventricular apical thin point		1/11		
Single umbilical artery	1/10			
Abdominal abnormalities			1/10	

CNVs, copy number variants; w, weeks; y, years.

In Table 3, 10 prenatally detected 1q12.1 microdeletion cases with detailed microarray results and ultrasound findings were collected (Figure 1C) (2, 10, 18–22). The gestational age was between 13 and 25 weeks. All cases varied in size and ranged from 317 kb to 1.89 Mb: class I deletion (5/10), class II deletion (2/10), TAR syndrome (1/10), and atypical 1q21.1 deletion (2/10). Among these deletions, 5 of 10 were parentally inherited, 3 of 10 were *de novo*, and 2 of 10 cases were not available. Pregnancy outcomes were as follows: five gave birth to a child or opted for ongoing pregnancy, three chose TOP, and two were unavailable. The pooled data from all fetuses presenting 1q21.1 microdeletion in the published literature and our study are listed in Table 4. Maternal age at diagnosis ranged from 26 to 34 years in the published literature and ranged from 18 to 42 years in our cases, with a mean age of 29 years. The gestational age at diagnosis ranged from 13 to 25 weeks in the published cases and from 16 to 29 weeks in our cases, with the mean gestational age of 19 and 20 weeks, respectively. The summarized frequencies of abnormal prenatal phenotypes in the literature and our study were as follows: increased NT (5/21), anomalies of the urinary system (4/21), cardiovascular abnormalities (3/21), nervous system abnormalities (2/21), oligohydramnios (2/21), skeletal dysplasia (1/21), fetal growth restriction (1/21), aberrant right subclavian artery (1/21), ventricular apical thin point (1/21), and single umbilical artery (1/21). Based on the findings mentioned above, we assumed that 1q21.1 deletions were closely associated with increased NT, anomalies of the urinary system, and cardiovascular abnormalities in prenatal ultrasound findings. It was noteworthy that the asymptomatic pregnant woman in our case 9 transmitted the 1q21.1 microdeletion to her two pregnancies. However, the first fetus showed VSD, while the second fetus showed no anomalies in ultrasonography, which indicated the incomplete penetrance of the 1q21.1 deletion in the prenatal setting. Meanwhile, diverse prenatal phenotypes of 1q21.1 deletions may be presented in the same family. In addition, TAR syndrome could be prenatally diagnosed by abnormal examination, mainly including bilateral radial hypoplasia/agenesis with or without humeral shortness and the presence of thumbs on both hands (23). No limb anomalies were observed except for increased NT in prenatal ultrasound findings for our case 1, which might indicate the phenotypic diversity of TAR syndrome in the prenatal setting. However, whether TAR syndrome should be added to the long list of genetic syndromes associated with increased NT should require more clinical evidence.

In Table 5, 10 prenatal 1q21.1 microduplication cases with detailed microarray results and ultrasound findings are reviewed (Figure 2C) (1, 6, 10, 15, 24, 25). The gestational age was between 11 and 27 weeks. The duplicated size ranged from 258 kb to 2.7 Mb: class I duplication (6/10), class II duplication (1/10), and 1q21.1 duplication involving the TAR region (3/10). Among these duplications, 5 of 10 were maternally inherited, 4 of 10 were *de novo*, and 1 of 10 were unavailable. Four cases opted for TOP, and the pregnancy outcomes of the remaining cases were unavailable. The pooled data from all fetuses presenting 1q21.1 microduplication in the published literature and our study are listed in Table 4. Maternal age at diagnosis ranged from 23 to 32 years in the published literature and from 23 to 45 years in our cases, with mean ages of 28 and 33 years, respectively. The gestational age at diagnosis ranged from 11 to 27 weeks in the published cases and ranged from 17 to 28 weeks in our cases, with a mean gestational age of 21 weeks. The summarized frequencies of abnormal prenatal phenotypes in the literature and our study were as follows: cardiovascular abnormalities (7/25), nasal bone dysplasia (5/25), increased NT (4/25), anomalies of the urinary system (2/25), cerebral ventriculomegaly (2/25), oligohydramnios (2/25), skeletal dysplasia (1/25), and abdominal abnormalities (1/25). In addition, Ji et al. inferred that nasal bone loss might be related to 1q21.1 duplication (1). Based on the findings mentioned above, we assumed that 1q21.1 duplications were closely correlated with cardiovascular abnormalities, nasal bone dysplasia, and increased NT in prenatal settings. In addition, cerebral ventriculomegaly observed in our study was not reported in prenatally detected 1q21.1 microduplication before, which might be associated with 1q21.1 duplication, but more evidence should be collected.

**Table 5:** Clinical data of fetuses presenting 1q21.1 microduplication detected by CMA in the published literature.

**No**.	**Age**	**Gravida and para**	**Gestational age (weeks)**	**Karyotype**	**Duplicated region**	**Duplicated size**	**Inheritance**	**CMA results (GRCh38)**	**Referred critical gene**	**Prenatal ultrasound findings**	**Pregnancy outcome**	**References**
10	29	G2P0	18+	46,XY	1q21.1	258 kb	*de novo*	1q21.1 (145601946-145809125) × 3	PDZK1, GPR89A, CD160, RNF115, GPR89C	Bilateral polycystic kidney; oligohydramnios; VSD	Not referred	(10) case 1
11	30	G2P1	24+	46,XX	1q21.1q21.2	1.34 Mb	*de novo*	1q21.1q21.2 (147004967-148348214) × 3	GJA5, GJA8	Absent nasal bone	TOP	(1) fetus 1
12	28	G1P0	25+	46,XY	1q21.1q21.2	1.35 Mb	Mat	1q21.1q21.2 (147004967-148354661) × 3	GJA5, GJA8	Duodenal atresia	TOP	(1) fetus 2
13	23	G2P0	26+	46,XY	1q21.1q21.2	2.69 Mb	*de novo*	1q21.1q21.2 (146348587-149562723) × 3	GJA5, GJA8	Absent nasal bone	TOP	(1) fetus 3
14	32	n.a.	20+	n.a.	1q21.1	2.6 Mb	Mat	1q21.1 (146194878-148342566) × 3	GJA5, PRKAB2	Cardiomyopathy, absent pulmonary valve	TOP at 22 w 6 d	(6)
15	n.a.	n.a.	n.a.	46,XN	1q21.1q21.2	1.71 Mb	*de novo*	1q21.1q21.2 (146066001-148342369) × 3	GJA5	VSD, pulmonary stenosis, persistent left superior vena cava	Not referred	(25)
16	n.a.	n.a.	n.a.	n.a.	1q21.1q21.2 22q11.21	0.9 Mb 2.7 Mb	n.a.	1q21.1q21.2 (147029796-147926447) × 3 22q11.21 (18857119-21181815) × 1	GJA5, GJA8	NT = 4.0 mm, VSD	n.a.	(24)
17	n.a.	n.a.	11+	n.a.	1q21.1	1.678 Mb	Mat	1q21.1 (145430980-148435812) × 3	PEX11B, RBM8A, POLR3GL HJV, GJA5, GJA8	NT = 3.5 mm; bladder, dilated tense; two vessel cord	n.a.	
18	n.a.	n.a.	27+	n.a.	1q21.1	559 kb	Mat	1q21.1 (145601946-146062451) × 3	PEX11B, RBM8A, POLR3GL, HJV	AV canal; oligohydramnios	n.a.	(15)
19	n.a.	n.a.	20+	n.a.	1q21.1	639 kb	Mat	1q21.1 (145601946-146062452) × 3	PEX11B, RBM8A, POLR3GL, HJV	Overlapping fingers-bilateral; elbow, fixed flexed-bilateral; knee, fixed extended-bilateral; thorax, other	n.a.	

CMA, chromosomal microarray analysis; LP, likely pathogenic; mat, maternal; n.a., not available; w, weeks; d, days; NT, nuchal translucency; P, pathogenic; TOP, termination of pregnancy; VOUS, variants of unknown significance; VSD, ventricular septal defect.

1q21.1 recurrent microduplication/microdeletion shares the same coordinates within the 1q21.1 region. Of the 26 fetuses in our study, 22 cases (10 1q21.1 deletions and 12 1q21.1 duplications) covered partial or the whole 1q21.1 recurrent microduplication/microdeletion region, in which 9 OMIM genes (*PRKAB2, FMO5, CHD1L, BCL9, ACP6, GJA5, GJA8, GPR89B*, and *NBPF24*) were located. Among them, the *GJA5* and *GJA8* genes are morbid genes associated with clinical diseases. As a critical candidate gene for the cardiac phenotype, *GJA5* encodes the gap junction protein connexin 40 (CX40), and its heterozygous mutations would lead to familial atrial fibrillation 11 (OMIM 614049) and atrial standstill (OMIM 108770) (3). *GJA5* might be responsible for the phenotypic specificity in congenital heart disease (CHD) resulting from 1q21.1 CNVs (26, 27). Its flanking gene, *GJA8* (OMIM 600897), encodes the gap junction protein connexin 50 (CX50), and its heterozygous mutation would result in autosomal dominant cataract 1, multiple types (OMIM 116200) (28). The abnormal expression of *GJA5* and *GJA8* has been closely associated with CHD (10). Cases 17, 25, and the first pregnancy of case 9 in our study presented VSD, which might be attributed to the losses and gains of *GJA5* and *GJA8* genes. The pooled data from all reviewed studies indicate that the losses and gains of the *GJA5* and *GJA8* genes were probably the most common genetic causes associated with 1q21.1 deletions or duplications. The *PRKAB2* gene, highly expressed in the right ventricular outflow tract and skeletal muscles, has been associated with schizophrenia (29–31). The *CHD1L* gene, implicated in chromatin remodeling, relaxation, and decatenation, plays a role in DNA damage response. Overexpression of *CHD1L* was discovered in the tetralogy of Fallot (TOF), double-outlet right ventricle, and infundibular pulmonary stenosis (32). In addition, *CHD1L* is regarded as a candidate gene for autism, ADHD, and congenital anomalies of the kidney and urinary tract (19, 31). Our case 25 presented TOF, which might be correlated with *CHD1L* to some degree, but the correlation between *CHD1L* and TOF still needs further investigation. The *BCL9* gene is proposed as a candidate gene for schizophrenia (33). Meanwhile, it is also involved in language deficits in patients with the 1q21.1 duplication (31). With current knowledge, there is not enough evidence to show that the functions and effects of the remaining OMIM genes have close correlations with the abnormal phenotypes observed in the prenatal setting.

Four fetuses carrying 1q21.1 deletions (case 1) and 1q21.1 duplications (cases 12-14) involved the TAR region. A total of 10 OMIM genes are located in the TAR region, including *CD160, RNF115, POLR3C, PIAS3, ITGA10, PEX11B, RBM8A, POLR3GL, TXNIP*, and *HJV*. The *RBM8A* gene encodes the exon-junction complex subunit member Y14, one of the four components of the exon-junction splicing complex, which plays a critical role in cellular functions (34). The first study revealing the correlation between heterozygous 1q21.1 microdeletion involving the *RBM8A* gene and TAR syndrome was reported in 2007 (35). Along with further study on TAR syndrome, it has been observed that 1q21.1 deletion is regarded as necessary but not sufficient to result in TAR syndrome. Other genetic alterations could be involved in the process. Most patients with TAR syndrome had compound heterozygous mutations, including a proximal 1q21.1 deletion spanning at least 200 kb involving the *RBM8A* gene and one of the two low-frequency SNPs in regulatory regions of the *RBM8A* gene (18). There is currently insufficient evidence that other genes are associated with TAR syndrome and the prenatal phenotypes observed in our study.

There are some limitations to our study. First, the enrolled subjects with 1q21.1 deletions and duplications were collected in a single center, and the total number was relatively small. In order to establish a more clear correlation between 1q21.1 deletions/duplications and prenatal phenotypes, multi-center collaboration should be adopted to enlarge the sample size in the future. Second, the current follow-up outcomes were acquired after birth, and all participants were still young. Although all subjects with 1q21.1 deletions and duplications were in a healthy state with no obvious abnormalities observed until the writing of this article, it is difficult to predict whether abnormal phenotypes will appear in the future. Thus, long-term follow-up should be guaranteed, including for autism, intellectual disability, ADHD, hearing impairments, seizures, cardiac disease, and motor difficulties (12). In addition, some cases carried VOUS CNVs in addition to 1q21.1 microdeletions or duplications, and whether these CNVs would have potential impacts still needed further investigation.

## 5. Conclusion

In conclusion, we described the clinical data and molecular findings in 26 prenatal cases aiming to investigate the correlation between 1q21.1 CNVs and prenatal phenotypes. Our study mainly focused on the prenatal phenotypes of 1q21.1 CNVs based on our findings and the published cases. In the prenatal setting, 1q21.1 microdeletions were associated with increased nuchal translucency (NT), anomalies of the urinary system, and cardiovascular abnormalities, while 1q21.1 microduplications were correlated with cardiovascular malformations, nasal bone dysplasia, and increased NT. In addition, cerebral ventriculomegaly might be correlated with 1q21.1 microduplications. More relevant studies are necessary for a better understanding of the prenatal phenotype–genotype correlation of 1q21.1 CNVs. For postnatal cases with 1q21.1 CNVs, regardless of whether ultrasound anomalies were observed or not during the pregnancy period, regular follow-up should be guaranteed till adulthood in case abnormal developmental behaviors may emerge.

## Data availability statement

The data presented in the study are deposited in the Gene Expression Omnibus repository, accession number GSE240611.

## Ethics statement

The study was approved by the Ethics Committee of the First Hospital of Jilin University. Written informed consent to participate was obtained for all the couples before collecting samples of amniotic fluid.

## Author contributions

FY obtained the clinical information, collected data from the literature, and wrote the manuscript. XY, YJ, and HZ performed the cytogenetic study and chromosomal microarray analysis on the amino fluid samples. SL, RL, and HZ conceived and designed the study, performed the final review, and editing of the manuscript. All authors have read and approved the final manuscript.
